# Quorum Sensing Regulation of the Two *hcp* Alleles in *Vibrio cholerae* O1 Strains

**DOI:** 10.1371/journal.pone.0006734

**Published:** 2009-08-24

**Authors:** Takahiko Ishikawa, Pramod Kumar Rompikuntal, Barbro Lindmark, Debra L. Milton, Sun Nyunt Wai

**Affiliations:** Department of Molecular Biology, Umeå University, Umeå, Sweden; Charité-Universitätsmedizin Berlin, Germany

## Abstract

**Background:**

The type VI secretion system (T6SS) has emerged as a protein secretion system important to several Gram-negative bacterial species. One of the common components of the system is Hcp, initially described as a hemolysin co-regulated protein in a serotype O17 strain of *Vibrio cholerae*. Homologs to *V. cholerae hcp* genes have been found in all characterized type VI secretion systems and they are present also in the serotype O1 strains of *V. cholerae* that are the cause of cholera diseases but seemed to have non-functional T6SS.

**Methodology/Principal Findings:**

The serotype O1 *V. cholerae* strain A1552 was shown to express detectable levels of Hcp as determined by immunoblot analyses using polyclonal anti-Hcp antiserum. We found that the expression of Hcp was growth phase dependent. The levels of Hcp in quorum sensing deficient mutants of *V. cholerae* were compared with the levels in wild type *V. cholerae* O1 strain A1552. The expression of Hcp was positively and negatively regulated by the quorum sensing regulators HapR and LuxO, respectively. In addition, we observed that expression of Hcp was dependent on the cAMP-CRP global transcriptional regulatory complex and required the RpoN sigma factor.

**Conclusion/Significance:**

Our results show that serotype O1 strains of *V. cholerae* do express Hcp which is regarded as one of the important T6SS components and is one of the secreted substrates in non-O1 non-O139 *V. cholerae* isolates. We found that expression of Hcp was strictly regulated by the quorum sensing system in the *V. cholerae* O1 strain. In addition, the expression of Hcp required the alternative sigma factor RpoN and the cAMP-CRP global regulatory complex. Interestingly, the environmental isolates of *V. cholerae* O1 strains that showed higher levels of the HapR quorum sensing regulator in comparison with our laboratory standard serotype O1 strain A1552 where also expressing higher levels of Hcp.

## Introduction

*Vibrio cholerae*, primarily known as the causal organism of the diarrheal disease cholera, is found as a free-living environmental organism within aquatic natural reservoirs and there are many different types distinguished by serological classification. Cholera is caused by the well studied O1 and O139 serotypes but recent findings with isolates of non-O1 non-O139 serotypes are providing new insights into the physiology and properties of *V. cholerae* as a versatile bacterium in different environments. *V. cholerae* has evolved several secretion systems to export toxins, enzymes, and other proteins necessary for the bacterial growth and survival in different environments and for bacteria-host interactions. The recent discoveries include the type VI secretion system (T6SS) that appears to be quite common in many different bacteria. Genes encoding a putative T6SS are present in nearly 25% of all sequenced Gram-negative bacterial species [1]. The T6SS has been implicated in virulence of *V. cholerae* [2], *Pseudomonas aeruginosa* [3], *Edwardsiella tarda* [4], [5], *Burkholderia* species [6], [7], [8], and *Aeromonas hydrophila* [9]. Earlier studies by Bladergroen et al. [10] suggested that the *imp* locus (impaired in nodulation) in *Rhizobium leguminosarum* may utilize an unconventional secretion system with characteristics of both types III and IV secretion systems and indicated an involvement of this locus in the secretion of a periplasmic protein to the extracellular environment. The work defining the *imp* locus in *R. leguminosarum* and pathogenesis of this bacteria has been concomitant with work on *V. cholerae* classical O1 strain O395, which started with the characterization of an IcmF homologue [11]. The expression of *icmF* in *V. cholerae* strain O395 was induced inside the host intestinal environment and the expression was correlated with motility of the organism. The *icmF* insertion mutant showed increased adherence to intestinal epithelial cells and increased conjugation frequency as a recipient [12].

It was also suggested that the *imcF* gene may be part of a secretion system that is capable of transferring a substrate across the outer membrane or might take part in the transport of effector molecules. The presence of genes for a phosphoprotein phosphatase and a Ser/Thr protein kinase adjacent to some of the *icmF* homologs also suggested the possible involvement in transport of effector molecules in signaling pathway [13]. The *V. cholerae* O1 *icmF* gene was identified as being induced *in vivo* in a rabbit model of infection [12], [14]. Further studies with the O37 serogroup *V. cholerae* revealed the role of the *V. cholerae* IAHP cluster as the new T6SS [15]. The *V. cholerae* strain V52, in contrast to the O1 strain, is capable of amoeba killing when plated with *Dictyostelium discoideum* [15]. Transposon mutagenesis of strain V52 identified a series of mutants that were attenuated for their virulence on *Dictyostelium*. The transposon mapping revealed a cluster of genes called *vas* for (virulence-associated secretion). Several Vas-secreted putative effector proteins lacking a hydrophobic N-terminal signal were also identified, and a double mutant lacking the Vas-secreted identical proteins Hcp (haemolysin co-regulated protein)-1 and Hcp-2 was found to be avirulent towards *D. discoideum* [15].

The gene cluster-encoded T6SS mediates the extracellular secretion of four distinct proteins (Hcp, VgrG-1, VgrG-2, and VgrG-3). Mutations in the *hcp* or the *vgrG-2* genes in the non-O1 non-O139 *V. cholerae* strain V52 attenuate cytotoxicity and block secretion of the other T6SS protein substrates suggesting that both of the Hcp and the VgrG proteins are T6SS machine components or involved in targeting the T6SS dependent substrates to be secreted outside the bacterial cells [2]. The functional significance of these genes was initially characterized in the non-O1 non-O139 *V. cholerae*, where the ability to perform secretion of Hcp and VgrG proteins into supernatant fluids was shown to be important as a defense against predation by *D. discoideum* [15]. Hcp assembles into hexameric ring structures that may form a regulated pore for secretion of putative effectors out of the bacterium [16]. VgrG proteins, which have homology to trimeric phage T4 tail spike proteins, are suggested to assemble into a trimer that associates with Hcp. It is proposed that VgrG proteins thus extend away from the bacterium to contact the eukaryotic cell membrane and transfer effectors directly to the target eukaryotic cell cytosol. The reciprocal requirement of Hcp and VgrG proteins for secretion suggests that Hcp and VgrG are secretion substrates that are transported through a putative core T6SS complex and could also comprise components of an extracellular portion of the T6SS apparatus that can then shear off from bacterial cells [2], [7].

Very little is known about the regulation of Hcp expression in *V. cholerae* apart from the initial finding that two genetic loci for Hcp are coregulated with the genes for hemolysin in the non-O1 non-O139 *V. cholerae* serotype O17 strain [17]. Mutating both of the *hcp* genes in the *V. cholerae* O17 strain genome did not affect the virulence of the pathogen in the infant mouse infection model [17]. It was also reported that in a survey of 18 different *V. cholerae* strains examined by Western blot analysis only 4 strains showed detectable levels of Hcp [17]. T6SS genes are also present in *V. cholerae* O1 strains but were regarded non-functional [2]. This prompted us to carry out more detailed analysis of the *hcp* gene expression in the typical O1 *V. cholerae* strain A1552. Many Gram-negative bacteria use a population density-dependent regulatory mechanism called quorum sensing (QS) to control the production of virulence factors during infection [18] QS plays an essential role in the pathogenesis of many bacterial pathogens of both plants and animals [18].

In this study we show that the transcription of the *hcp* genes in the O1 strain is growth phase dependent and subject to control mediated by the cyclic AMP receptor protein CRP, an alternative sigma factor RpoN, and the quorum sensing dependent regulator HapR.

## Materials and Methods

### Bacterial strains, culture conditions and plasmids

The bacterial strains and plasmids used in this study are listed in Table 1. Bacterial strains were grown overnight at 37°C with shaking in Luria-Bertani (LB) broth supplemented, as appropriate, with kanamycin (50 µg/ml), or carbenicillin (100 µg/ml). The Δ*hcp1*, Δ*hcp2*, Δ*hcp1 Δhcp2, Δcrp, Δcya, ΔcqsA, ΔluxS, ΔhapR, ΔluxO, ΔrpoN, and ΔrpoS* mutants were constructed by making deletions of the entire reading frame using procedures that have been described previously [19], [20]. Oligonucleotide primers used are listed in Table 2.

**Table 1:** Bacterial strains and plasmids.

Strains/Plasmids	Relevant Genotype/Phenotype	Reference/Source
**Bacteria**		
*E. coli* DH5α	F^−^, ø80d*lacZ*ΔM15, Δ(*lacZYA-argF*)U169, *deoR*, *recA*1, *endA*1, *hsdR*17(rk^−^, mk^+^), *phoA*, *supE*44, λ^−^, *thi*-1, *gyrA*96, *relA*1	[34]
*E. coli* SM10λpir	*thi thr leu tonA lacY supE recA*::RP4-2 Tc::Mu Km λpir	[35]
*V. cholerae* A1552	O1 El Tor, Inaba, Rif^R^	[36]
*V. cholerae* A1552*Δhcp1*	*Δhcp1* derivative of A1552	This study
*V. cholerae* A1552*Δhcp2*	*Δhcp2* derivative of A1552	This study
*V. cholerae* A1552*Δhcp1,2*	*Δhcp1,2* derivative of A1552	This study
*V. cholerae* A1552*ΔhapR*	*ΔhapR* derivative of A1552	This study
*V. cholerae* A1552*ΔrpoS*	*ΔrpoS* derivative of A1552	This study
*V. cholerae* A1552*ΔrpoN*	*ΔrpoN* derivative of A1552	This study
*V. cholerae* A1552*Δcrp*	*Δcrp* derivative of A1552	This study
*V. cholerae* 1552*Δcrp/*pHA7	complementation of A1552*Δcrp*	This study
*V. cholera* 1552*Δcrp/*pBR322	A1552*Δcrp* with vector control	This study
*V. cholerae* A1552*Δcya*	*Δcya* derivative of A1552	This study
*V. cholerae* A1552*ΔluxO*	*ΔluxO* derivative of A1552	This study
*V. cholerae* A1552*ΔluxS*	*ΔluxS* derivative of A1552	This study
*V. cholerae* A1552*ΔcqsA*	*ΔcqsA* derivative of A1552	This study
*V. cholerae* A1552*Δhfq*	*Δhfq* derivative of A1552	[37]
*V. cholerae* V5:/04	non-O1 non-O139 clinical isolate (2004)	Swedish Institute of Infectious Diseases
*V. cholerae* C6706	O1 El Tor, Inaba, Str^R^	[38]
*V. cholerae* AJ3	O1 Environmental isolate (1981)	Ryukyu University
*V. cholerae* AJ5	O1 Environmental isolate (1981)	Ryukyu University
*V. cholerae* CB4	O1 Classical Inaba (1982)	Ryukyu University
*V. cholerae* 86B1	O1 Classical Ogawa (1986)	Ryukyu University
**Plasmids**		
pBR322	Cb^R^ cloning vector plasmid	[39]
pHA7	pBR322 based *crp* expression plasmid	[40]
pGEM-TEasy	Cb^R^ TA-cloning vector plasmid	Promega®
pCVD442	Cb^R^ positive selection suicide vector plasmid	[41]
pKAS32	Cb^R^ positive selection suicide vector plasmid	[42]
pTYB1	Cb^R^, expression vector plasmid	New England iolabs®
p*Δhcp1*	pCVD442-based suicide plasmid for generating *Δhcp1*	This study
p*Δhcp2*	pCVD442-based suicide plasmid for generating *Δhcp2*	This study
p*ΔrpoS*	pCVD442-based suicide plasmid for generating *ΔrpoS*	[43]
p*ΔrpoN*	pCVD442-based suicide plasmid for generating *ΔrpoN*	This study
p*ΔhapR*	pKAS32-based suicide plasmid for generating *ΔhapR*	[19]
p*Δcrp*	p KAS32-based suicide plasmid for generating *Δcrp*	This study
p*Δcya*	p KAS32-based suicide plasmid for generating *Δcya*	This study
p*ΔluxO*	p KAS32-based suicide plasmid for generating *ΔluxO*	[20]
p*ΔluxS*	p KAS32-based suicide plasmid for generating *ΔluxS*	[20]
p*ΔcqsA*	p KAS32-based suicide plasmid for generating *ΔcqsA*	[20]

**Table 2:** Primers used in this study.

Primer	Sequence	Source
HCP1-A	5′ CGCTCTAGAGGGTGGCTTGCTGCTCGATAT3′	This study
HCP1-B	5′CCCATCCACTATAAACTAACAGCCGATAGAGTCAGCAGTACA3′	This study
HCP1-C	5′TGTTAGTTTATAGTGGATGGGGCGTAATTTACGCTGCGAGAA3′	This study
HCP1-D	5′ CGCTCTAGACCCATCTCTTGCAGCAAGATA3′	This study
HCP2-A	5′CGCTCTAGACCCATCTCTTGCAGCAAGATA3′	This study
HCP2-B	5′CCCATCCACTATAAACTAACAGCCTGCAGTGATAAGACCCT3′	This study
HCP2-C	5′TGTTAGTTTATAGTGGATGGGGCGTAATTTACGCTGCGAGAA3′	This study
HCP2-D	5′ CGCTCTAGACCCATCTCTTGCAGCAAGATA3′	This study
RpoN-A	5′ CGCTCTAGACTCTCGGTCGAAGACAACATC3′	This study
RpoN-B	5′CCCATCCACTATAAACTAACACGGTTTCATGCAGTAATGGA3′	This study
RpoN-C	5′TGTTAGTTTATAGTGGATGGGCGCCTGCTATAGGCCTAAACT3′	This study
RpoN-D	5′ CGCTCTAGAGGCTATCCCGTTACCAATAC3′	This study
CRP-A	5′ CGCGAATTCAACCCACTCAATGCCAAAGGC3′	This study
CRP-B	5′CCCATCCACTATAAACTAACATGGATCGGT3′TTGAGGTTTACC3′	This study
CRP-C	5′TGTTAGTTTATAGTGGATGGGTCTCTGCCCACGGTAAAACCA3′	This study
CRP-D	5′ CGCGATATCTGTATGGCGGTCGCTTATGGA3′	This study
Cya-A	5′ TCGGCGTCTCGGTCATTATGA3′	This study
Cya-E	5′CCCATCCACTATAAACTAACAGGCTTCAACACCCTGACTTTTCGC3′	This study
Cya-F	5′TGTTAGTTTATAGTGGATGGGGGCTCGCCTTTACTGATGCTC3′	This study
Cya-D	5′ ATAATCGCACCACGATTAGGA3′	This study

### SDS-PAGE and Immunoblot analyses

To determine the levels of protein expression, the bacterial strains were grown in LB medium and the samples were taken at different growth phases (i. e. OD_600 nm_ 1.0, 2.0, 3.0, and overnight) and centrifuged at 14,000 g×2 min. The bacterial cell pellets were suspended in 20 mM Tris-HCl pH 8.0 buffer and used for SDS-PAGE and immunoblot analyses. The protein samples were separated by sodium dodecyl sulfate-13% polyacrylamide gel electrophoresis (SDS-PAGE) [21]. Western blot analyses were performed as described [22] using anti-Hcp polyclonal antiserum (this study), anti-CRP polyclonal antiserum [23], anti-RpoN antiserum (this study) and anti-HapR antiserum [24]. The immunoblot detection was done by using the ECL+ chemiluminescence system (GE Healthcare, United Kingdom).

### Anti-Hcp polyclonal antiserum preparation

Anti-Hcp polyclonal antibodies were raised in rabbits using the following method. Briefly, *V. cholerae* non-O1 non-O139 strain V5 produced a large amount of Hcp protein that was efficiently secreted into the culture supernatants. The secreted 28 kDa protein was confirmed by the Mass Spectrometric analysis as the Hcp of *V. cholerae* strains (data not shown). The Hcp was excised from the SDS gel and eluted from the gel. The eluted Hcp protein was used for immunization of the rabbits.

### Anti-RpoN polyclonal antiserum preparation

The *rpoN* gene was amplified by PCR and cloned into pTYB1 using *Nde*I and *Sap*I restriction enzyme sites. The RpoN protein was purified using the IMPACT T7 system from New England Biolabs described by the manufacturer. Using the purified RpoN protein, polyclonal rabbit antiserum was produced by AgriSera AB, Sweden.

### In vivo protein stability experiment

The stability of the Hcp proteins was examined using a procedure as described earlier [25] with some modifications. Protein stability was monitored after the protein synthesis had been inhibited by the addition of 25 µg/ml chloramphenicol to bacterial cultures grown to OD_600nm_ at 2.0 in LB medium at 37°C. Samples to be analyzed by western blotting were removed at indicated time points: 0; 15; 30; 60 min; 3 and 6 hrs after addition of chloramphenicol.

## Results

### Growth phase dependent levels of intracellular Hcp in *V. cholerae* O1 strain A1552

**Figure 1:**
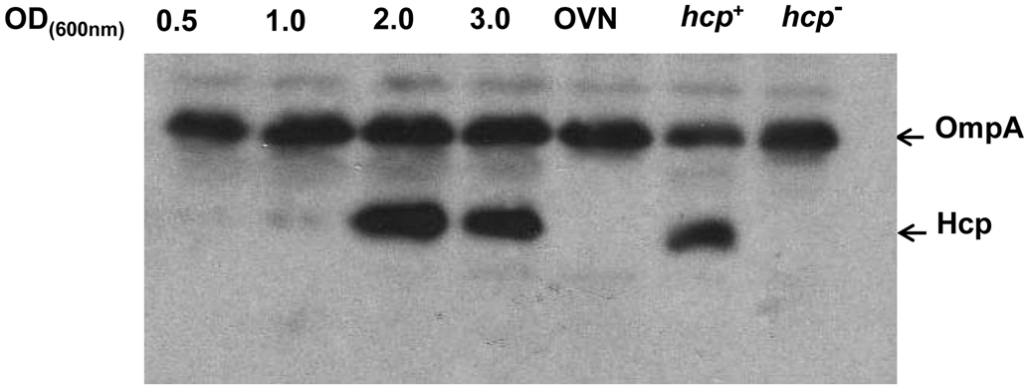
Hcp levels in *V. cholerae* O1 wild type strain A1552 at different growth phases. Bacteria were grown as described in Materials and Methods and the whole cell lysate samples were taken at different optical density. Immunoblot analysis was performed with an anti-Hcp antiserum that also contained antibodies recognizing the OmpA major outer membrane protein as confirmed by analysis of *ompA* mutant *V. cholerae* (data not shown).

Earlier it was reported that *V. cholerae* O1 and O139 strains also carry *hcp* and *vas* genes in their genomes but *e.g*. the O1 strain N16961 was unable to secrete Hcp into the culture supernatant indicating that the T6SS was not expressed or was non-functional in the O1 serotype [15]. As an initial study we tested the effect of bacterial growth phase on the expression of Hcp in *V. cholerae* O1 strain A1552. The bacteria were grown in a standard LB medium and the level of Hcp was measured at different growth phases by immunoblot analyses. We detected expression of Hcp at OD_600_ 1.0 and the highest expression was observed at OD_600_ 2.0 (Fig. 1). Interestingly, the Hcp band was not detectable in whole cell samples of overnight grown bacteria. Similar to what was reported for the O1 strain N16961 [15] there was no detectable secreted Hcp in culture supernatants from *V. cholerae* O1 strain A1552 at any of the different growth phases or in the late stationary phase after over night incubation (data not shown).

**Figure 2:**
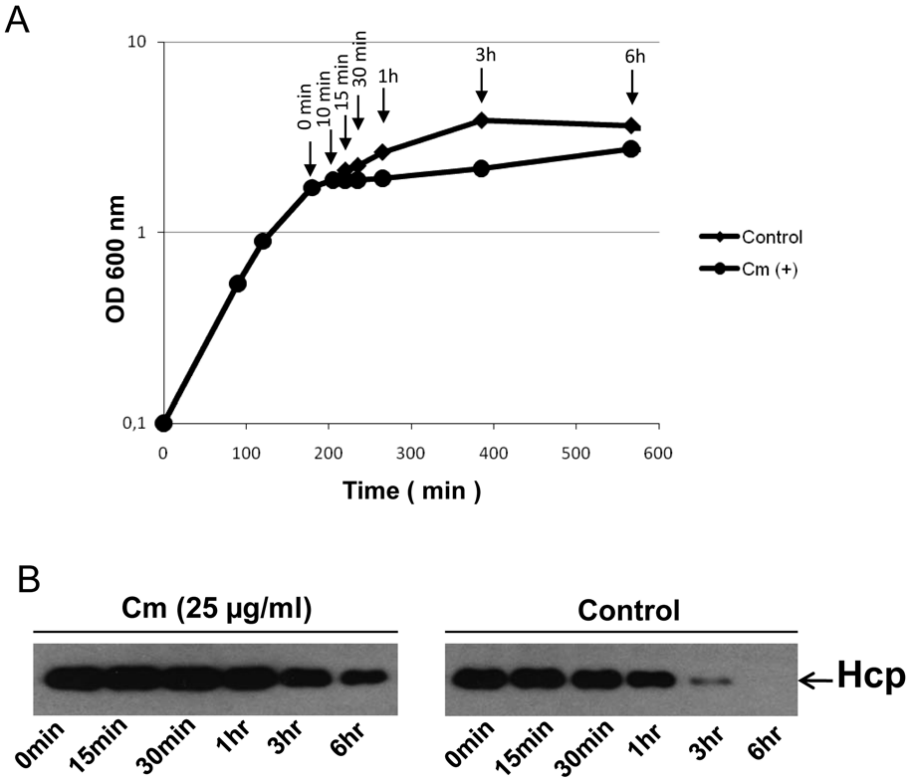
Analysis of Hcp stability in *V. cholerae* O1 wild type strain A1552. (A) The growth curve and time of sampling for Hcp analyses of *V. cholerae* wild type strain A1552 with and without chloramphenicol (Cm) treatment. The bacterial cells were grown to OD 2.0 and 25 µg/ml Cm was added. Arrows indicate the time points of sampling for immunoblot analysis after the addition of Cm. (B) Immunoblot analysis of the stability of Hcp in *V. cholerae* wild type strain A1552. The samples were taken at different time points after the addition of Cm (for the test sample). For the control experiment, the samples were taken at different time points during normal growth of bacteria. The vertical arrows show the time points when the samples were taken.

The apparent lack of Hcp in bacteria harvested from the stationary phase after overnight incubation might be due to an intracellular proteolytic degradation of the Hcp in late stationary phase and/or that the expression was totally shut off in the late stationary phase. Nothing was known about the possible degradation or turn over of Hcp in the bacterium. We therefore performed an experiment to monitor the stability of Hcp in *V. cholerae* wild type strain A1552 after inhibiting the total protein synthesis by addition of chloramphenicol into the culture medium as described in Materials and Methods (Fig. 2A&B). The Hcp level appeared stable during at least one hour after the total protein synthesis was inhibited and at least half of the protein remained after three hours suggesting that there was not much degradation or turn over of Hcp at that growth stage (Fig. 2B). In the control culture the level of Hcp was almost the same throughout the late exponential phase of bacterial growth. However, the Hcp level was reduced about 10-fold when the bacteria entered into the early stationary phase. In the late stationary phase (6 hr samples) the Hcp level was totally abolished in the control culture. The results suggested that the absence of Hcp in the bacteria of an overnight culture would be due to both loss of expression and to an increased turnover by proteolytic degradation presumably by some stationary phase expressed protease(s).

The *V. cholerae* O1 strain A1552 has two *hcp* genes separately located in the two *V. cholerae* chromosomes. Mutant derivatives with deletion of either of the two loci still expressed Hcp in a growth phase dependent manner from the intact locus as confirmed by immunoblot analysis (data not shown). The complete disappearance of Hcp in the stationary phase as judged by the results from the immunoblot analyses (Fig. 1 and 2) showed that there was loss of expression from both *hcp* loci and it appeared that expression was quite coordinated.

### Quorum sensing regulators HapR *and* LuxO influence the expression of Hcp in *V. cholerae* O1 strain A1552

**Figure 3:**
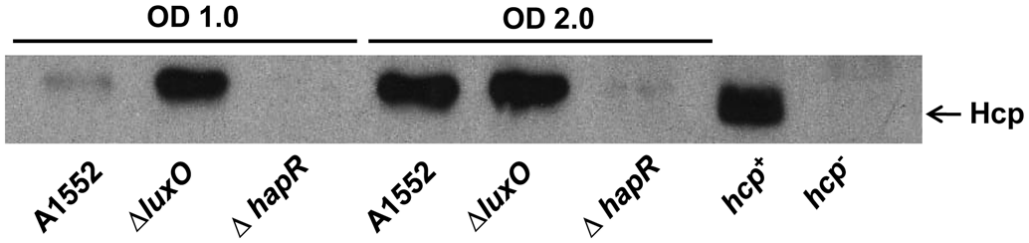
The effect of Δ*hapR* and Δ*luxO* mutations on Hcp levels in *V. cholerae* strain A1552. The whole cell lysate samples were taken at OD 1.0 and OD 2.0 and immunoblot analysis was performed using anti-Hcp polyclonal antiserum.

In order to analyze further how the expression of Hcp might be regulated in the growth phase dependent manner, we compared the level of Hcp in wild type *V. cholerae* strain A1552 with those of different global regulator mutant strains. We first tested the levels of Hcp expression in quorum sensing (QS) regulatory mutants since the QS is the main growth phase mediated regulatory system in *V. cholerae*. As shown in Fig. 3, the expression of Hcp was strongly reduced in the *hapR* mutant. Furthermore, the level of Hcp was clearly increased at an earlier growth phase (OD 1.0) in the *luxO* mutant *V*. *cholerae* derivative when compared to the wild type strain A1552. Our findings suggested that the expression of Hcp in the *V. cholerae* O1 strain was positively regulated by the quorum sensing regulator HapR and negatively regulated by the regulator LuxO.

### The role of up-stream quorum sensing regulators in expression of the Hcp

**Figure 4:**
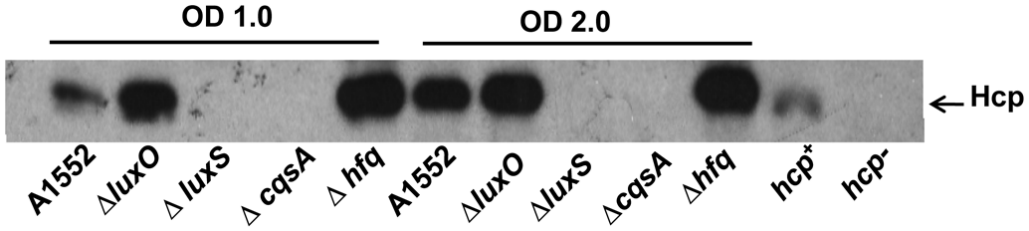
Hcp levels in different quorum sensing regulatory system of *V. cholerae* strain A1552. The samples were taken at OD 1.0 and OD 2.0 from wild type *V. cholerae* O1 strain A1552 and its quorum sensing regulator mutants and immunoblot analyses were done using anti-Hcp antiserum

We next tested the levels of Hcp in autoinducer synthesizer mutants such as Δ*cqsA* and Δ*luxS* and in a mutant defective in one of the master regulatory components of the quorum sensing system, the *hfq* gene. As shown in Fig. 4, in the Δ*hfq* mutant the expression of Hcp was increased at the earlier growth phase to the same extent as in the case of the Δ*luxO* mutant where the level of HapR also was expected to be up-regulated. In contrast, the level of Hcp was reduced in the Δ*cqsA* and Δ*luxS* mutants where the level of the *hapR* expression was expected to be reduced. The results were all consistent with our suggestion that expression of a functional *hapR* gene was essential for the expression of the *hcp* genes in the *V. cholerae* O1 strain.

**Figure 5:**
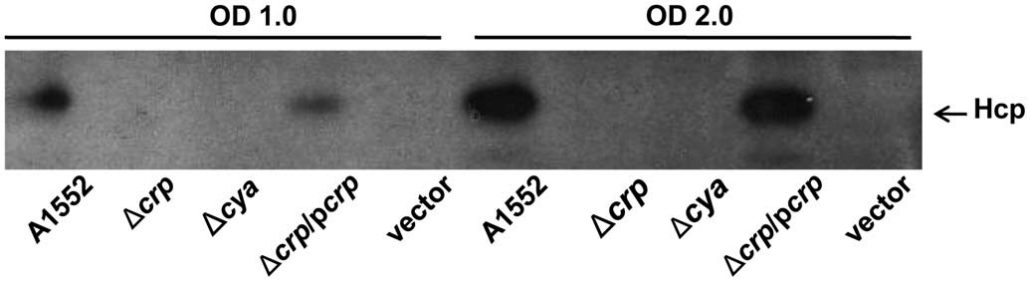
Hcp levels in Δ*crp* and Δ*cya* mutants of *V. cholerae* strain 1552. Immunoblot analysis of Hcp levels in whole cell lysates of *V. cholerae* O1 wild type strain A1552, Δ*crp*, Δ*cya*, Δ*crp*/p*crp*, and Δ*crp*/vector control strains. The whole cell lysates were taken at OD 1.0 and OD 2.0 and immunoblot was done using anti-Hcp antiserum.

As it was reported that the carbon catabolite repressor, CRP (cAMP-receptor protein) is required for the biosynthesis of cholera autoinducer 1 (CAI-1) and affects the expression of multiple HapR- regulated genes [26], we analyzed whether the expression of the Hcp would also be regulated by CRP and cAMP. We compared the levels of the Hcp expression in wild type *V. cholerae* strain A1552 and its *crp* mutant by immunoblot analyses. The Hcp protein was not detectable in the Δ*crp* mutant and Hcp expression was fully complemented when we tested the strain containing a *crp* ^+^ allele in trans (Fig. 5). As the adenylate cyclase enzyme encoded by the *cya* gene catalyzes formation of cAMP, which is indispensable for the function of CRP, we also constructed a *cya* deficient mutant and analyzed the level of Hcp. As expected, the expression of Hcp was abolished in the Δ*cya* mutant (Fig. 5). These results indicate that in addition to the quorum-sensing system regulation, the metabolic regulators may also affect the expression level of Hcp.

**Figure 6:**
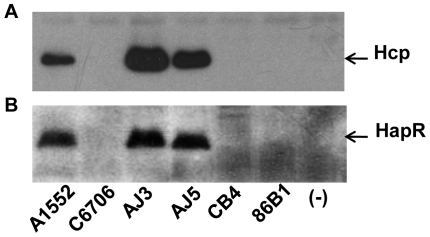
Levels of Hcp and HapR in different *V. cholerae* O1 isolates. (A) Immunoblot analysis of the expression of the Hcp in different *V. cholerae* O1 isolates was performed using anti-Hcp antiserum. (B) Detection of the HapR protein in same samples used for the detection of the Hcp. Samples were taken at OD 2.0. (-); negative controls for Hcp (Δ*hcp*1,2) and HapR (Δ*hapR*)

Furthermore, a quorum sensing and HapR-mediated regulation of Hcp was also suggested by results obtained with other *V. cholerae* O1 strains. As shown in Fig. 6A and B, the expression of Hcp was correlating to the HapR status of the different *V. cholerae* O1 isolates (A1552 & C6706; O1 El Tor, AJ3 & AJ5; O1 environmental, CB4 & 86B1; O1 Classical). Interestingly, the AJ3 and AJ5 strains, the environmental isolates of serotype O1 *V. cholerae* strains, showed even higher levels of Hcp expression than our standard strain A1552.

### RpoN (σ^54^) is essential for the expression of Hcp in the *V. cholerae* O1 strain A1552

**Figure 7:**
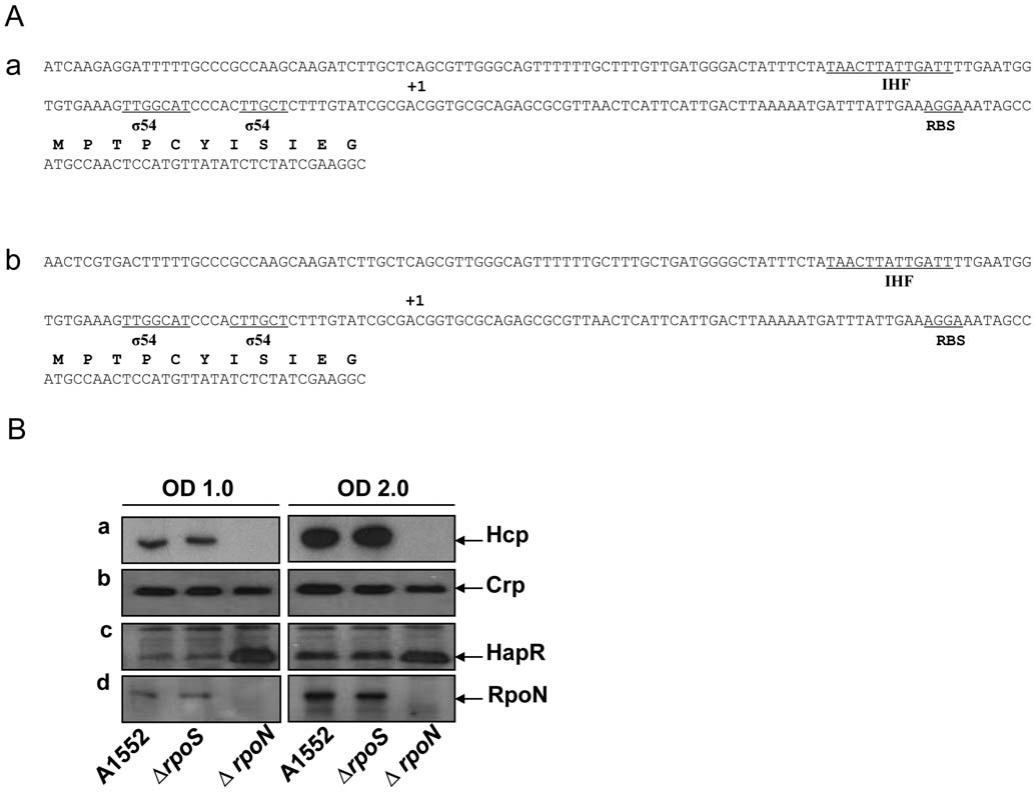
The *hcp*1 and *hcp*2 loci in *V. cholerae* strain A1552 and levels of global regulators. (A) DNA sequences of the promoter regions and part of open reading frames of *hcp*1 (a) and *hcp*2 (b) genes in *V. cholerae* O1 strain A1552. A potential ribosome binding sites (RBS), IHF binding sites (IHF), and sigma 54 binding sites (σ^54^) are underlined. The position corresponding to reported transcriptional start points in a serotype O17 *V. cholerae* strain [17] are labeled as +1. The amino acid sequences of the ORFs are also shown above the nucleotide sequences in bold type. (B) The levels of Hcp, CRP, HapR, and RpoN in the *V. cholerae* O1 wild type strain. Immunoblot analyses of whole cell extracts from *V. cholerae* O1 wild type strain A1552 at OD 1.0 and 2.0 was performed using anti-Hcp, anti-CRP, and anti-HapR polyclonal antisera.

Next we considered whether Hcp expression might also be regulated by common alternative sigma factors of Gram-negative bacteria. The most likely positions of the *hcp*1 and *hcp*2 promoters in strain A1552, as determined by sequence analysis, included the (-12/-24) σ^54^ consensus sequences in both cases (Fig. 7A). On the basis of the sequence data analysis, we performed genetic experiments to directly test the prediction that the two *hcp* promoters would be σ^54^ dependent. We constructed in-frame deletion mutations in the *rpoN* and *rpoS* alternative sigma factor genes of *V*. *cholerae* O1 strain A1551 and monitored the expression of Hcp in these mutants by immunoblot analyses. As shown in Fig. 7B: the expression of Hcp was completely abolished in the *rpoN* mutant whereas there was no significant change in the *rpoS* mutant (Fig. 7B; panel a). The results indicated that the RpoN (σ^54^) sigma factor would be essential for the expression of Hcp from either of the two loci in strain A1552. Our finding are also giving support to the previous prediction of a potential, but unconfirmed, σ^54^ binding sequence in the promoter regions of the *hcp* genes in *V. cholerae* non-O1 strains [17].

We also investigated the expression levels of the CRP, HapR and RpoN proteins in the Δ*rpoS* and Δ*rpoN* derivatives by immunoblot analyses using the same samples that were used for the detection of Hcp (Fig. 7). The HapR levels were increased in the Δ*rpoN* mutant at both OD 1.0 and 2.0 (Fig. 7C), in agreement with the previous reports that the *hapR* gene expression is negatively regulated by the RpoN sigma factor through four small RNAs, Qrrs, and Hfq in the quorum-sensing regulatory pathway of *V. cholerae* [27], [28]. Furthermore, this result supports our conclusion that RpoN would be directly required for *hcp* gene expression since in the Δ*rpoN* mutant the expression of Hcp was totally abolished despite the fact that in this mutant the level of HapR was increased.

## Discussion

**Figure 8:**
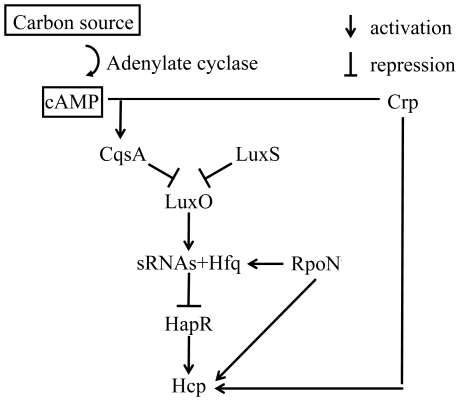
Regulation of Hcp expression in the *V. cholerae* O1 strain A1552. A schematic summary of the involvement of different regulators in the growth phase dependent expression of Hcp in strain A1552.

In this study we obtained evidence that there was expression of the T6SS substrate Hcp in *V. cholerae* O1 strain A1552 and we found that expression was strictly controlled by the quorum sensing regulatory system, the cAMP-CRP global metabolic regulator complex, and the alternative sigma factor RpoN as illustrated in Fig. 8. We monitored the expression of Hcp at different growth phases and observed that it was growth phase dependent and the maximum expression level was observed at OD 2.0. The Hcp was not detectable after the prolonged incubation in over-night growth cultures. However, as was pointed out earlier, the O1 serotype *V. cholerae* did not appear able to secrete Hcp and it was kept intracellular in the bacteria. It remains to be clarified when and how the complete T6SS determinant might be induced and functioning for protein secretion in the *V. cholerae* O1 strains. Our findings that the two *hcp* loci are expressed in the serotype O1 strains in a highly coordinated and regulated fashion suggest that these genes are functional and that Hcp may play a role in the bacterial growth and maintenance.

Bacterial pathogens use various protein secretion systems to deliver virulence effectors into hosts to cause diseases. The type III secretion system islands [29] and the recently identified type VI secretion system [15] are also virulence factors that are present in clinical and environmental isolates of different *V. cholerae* serotypes. Most secreted products of such gene clusters are associated with toxicity for eukaryotic cells and therefore may play a role in human disease or other pathobiological interactions with host or predatory environmental organisms. Protein secretion systems are generally precisely regulated by various global regulators to respond to various environmental changes or stresses. Regulation of the T6SS in *P. aeruginosa* is complex; the system is stringently regulated post-transcriptionally by the Gac/Rsm pathway and post-translationally by threonine phosphorylation [3], [16], [30]. There is also some precedence for quorum sensing control of T6SS in bacteria with an active secretion system. The VgrG ortholog ECA3427 is secreted by *Pectobacterium atrosepticum* [31] and expression of five VgrG orthologs and various other T6SS components was recently shown to be regulated by quorum sensing in plant host tissues or after exposure to host extracts [31], [32], [33] The expression level of four *P. atrosepticum hcp* homologs was clearly upregulated when plant extract was added to the medium and suggested that the expression of Hcp was under the influence of environmental signals [31]. The regulatory features of T6SS suggest that an optimal timing of T6SS gene expression is necessary to ensure correct function. Our characterization of the Hcp expression in serotype O1 *V. cholerae* should aid and prompt further studies of how Hcp in these human pathogenic strains may play some role in the bacterial life style. In particular it will be of interest to find out if there are conditions where Hcp would be actively secreted by the T6SS of the serotype O1 *V. cholerae* strains.

## References

[1] Bingle LE, Bailey CM, Pallen MJ (2008). Type VI secretion: a beginner's guide.. Curr Opin Microbiol.

[2] Pukatzki S, Ma AT, Revel AT, Sturtevant D, Mekalanos JJ (2007). Type VI secretion system translocates a phage tail spike-like protein into target cells where it cross-links actin.. Proc Natl Acad Sci U S A.

[3] Mougous JD, Cuff ME, Raunser S, Shen A, Zhou M (2006). A virulence locus of *Pseudomonas aeruginosa* encodes a protein secretion apparatus.. Science.

[4] Rao PS, Yamada Y, Tan YP, Leung KY (2004). Use of proteomics to identify novel virulence determinants that are required for *Edwardsiella tarda* pathogenesis.. Mol Microbiol.

[5] Zheng J, Leung KY (2007). Dissection of a type VI secretion system in *Edwardsiella tarda*.. Mol Microbiol.

[6] Aubert DF, Flannagan RS, Valvano MA (2008). A novel sensor kinase-response regulator hybrid controls biofilm formation and type VI secretion system activity in *Burkholderia cenocepacia.*. Infect Immun.

[7] Pilatz S, Breitbach K, Hein N, Fehlhaber B, Schulze J (2006). Identification of *Burkholderia pseudomallei* genes required for the intracellular life cycle and in vivo virulence.. Infect Immun.

[8] Schell MA, Ulrich RL, Ribot WJ, Brueggemann EE, Hines HB (2007). Type VI secretion is a major virulence determinant in *Burkholderia mallei*.. Mol Microbiol.

[9] Suarez G, Sierra JC, Sha J, Wang S, Erova TE (2008). Molecular characterization of a functional type VI secretion system from a clinical isolate of *Aeromonas hydrophila*.. Microb Pathog.

[10] Bladergroen MR, Badelt K, Spaink HP (2003). Infection-blocking genes of a symbiotic *Rhizobium leguminosarum* strain that are involved in temperature-dependent protein secretion.. Mol Plant Microbe Interact.

[11] Das S, Chaudhuri K (2003). Identification of a unique IAHP (IcmF associated homologous proteins) cluster in *Vibrio cholerae* and other proteobacteria through in silico analysis.. In Silico Biol.

[12] Das S, Chakrabortty A, Banerjee R, Chaudhuri K (2002). Involvement of in vivo induced *icmF* gene of *Vibrio cholerae* in motility, adherence to epithelial cells, and conjugation frequency.. Biochem Biophys Res Commun.

[13] Mukhopadhyay S, Kapatral V, Xu W, Chakrabarty AM (1999). Characterization of a Hank's type serine/threonine kinase and serine/threonine phosphoprotein phosphatase in *Pseudomonas aeruginosa*.. J Bacteriol.

[14] Das S, Chakrabortty A, Banerjee R, Roychoudhury S, Chaudhuri K (2000). Comparison of global transcription responses allows identification of *Vibrio cholerae* genes differentially expressed following infection.. FEMS Microbiol Lett.

[15] Pukatzki S, Ma AT, Sturtevant D, Krastins B, Sarracino D (2006). Identification of a conserved bacterial protein secretion system in *Vibrio cholerae* using the *Dictyostelium* host model system.. Proc Natl Acad Sci U S A.

[16] Mougous JD, Gifford CA, Ramsdell TL, Mekalanos JJ (2007). Threonine phosphorylation post-translationally regulates protein secretion in *Pseudomonas aeruginosa.*. Nat Cell Biol.

[17] Williams SG, Varcoe LT, Attridge SR, Manning PA (1996). *Vibrio cholerae* Hcp, a secreted protein coregulated with HlyA.. Infect Immun.

[18] Camilli A, Bassler BL (2006). Bacterial small-molecule signaling pathways.. Science.

[19] Vaitkevicius K, Lindmark B, Ou G, Song T, Toma C (2006). A *Vibrio cholerae* protease needed for killing of *Caenorhabditis elegans* has a role in protection from natural predator grazing.. Proc Natl Acad Sci U S A.

[20] Zhu J, Miller MB, Vance RE, Dziejman M, Bassler BL (2002). Quorum-sensing regulators control virulence gene expression in *Vibrio cholerae*.. Proc Natl Acad Sci U S A.

[21] Laemmli UK (1970). Cleavage of structural proteins during the assembly of the head of bacteriophage T4.. Nature.

[22] Towbin H, Staehelin T, Gordon J (1979). Electrophoretic transfer of proteins from polyacrylamide gels to nitrocellulose sheets: procedure and some applications.. Proc Natl Acad Sci U S A.

[23] Johansson J, Balsalobre C, Wang SY, Urbonaviciene J, Jin DJ (2000). Nucleoid proteins stimulate stringently controlled bacterial promoters: a link between the cAMP-CRP and the (p)ppGpp regulons in *Escherichia coli*.. Cell.

[24] Joelsson A, Kan B, Zhu J (2007). Quorum sensing enhances the stress response in *Vibrio cholerae*.. Appl Environ Microbiol.

[25] Geuskens V, Mhammedi-Alaoui A, Desmet L, Toussaint A (1992). Virulence in bacteriophage Mu: a case of trans-dominant proteolysis by the *Escherichia coli* Clp serine protease.. Embo J.

[26] Liang W, Pascual-Montano A, Silva AJ, Benitez JA (2007). The cyclic AMP receptor protein modulates quorum sensing, motility and multiple genes that affect intestinal colonization in *Vibrio cholerae*.. Microbiology.

[27] Lenz DH, Mok KC, Lilley BN, Kulkarni RV, Wingreen NS (2004). The small RNA chaperone Hfq and multiple small RNAs control quorum sensing in *Vibrio harveyi* and *Vibrio cholerae*.. Cell.

[28] Lenz DH, Bassler BL (2007). The small nucleoid protein Fis is involved in *Vibrio cholerae* quorum sensing.. Mol Microbiol.

[29] Tam VC, Serruto D, Dziejman M, Brieher W, Mekalanos JJ (2007). A type III secretion system in *Vibrio cholerae* translocates a formin/spire hybrid-like actin nucleator to promote intestinal colonization.. Cell Host Microbe.

[30] Goodman AL, Kulasekara B, Rietsch A, Boyd D, Smith RS (2004). A signaling network reciprocally regulates genes associated with acute infection and chronic persistence in *Pseudomonas aeruginosa*.. Dev Cell.

[31] Mattinen L, Nissinen R, Riipi T, Kalkkinen N, Pirhonen M (2007). Host-extract induced changes in the secretome of the plant pathogenic bacterium *Pectobacterium atrosepticum.*. Proteomics.

[32] Liu H, Coulthurst SJ, Pritchard L, Hedley PE, Ravensdale M (2008). Quorum sensing coordinates brute force and stealth modes of infection in the plant pathogen *Pectobacterium atrosepticum*.. PLoS Pathog.

[33] Mattinen L, Somervuo P, Nykyri J, Nissinen R, Kouvonen P (2008). Microarray profiling of host-extract-induced genes and characterization of the type VI secretion cluster in the potato pathogen *Pectobacterium atrosepticum*.. Microbiology.

[34] Hanahan D (1983). Studies on transformation of *Escherichia coli* with plasmids.. J Mol Biol.

[35] Miller VL, Mekalanos JJ (1988). A novel suicide vector and its use in construction of insertion mutations: osmoregulation of outer membrane proteins and virulence determinants in *Vibrio cholerae* requires *toxR*.. J Bacteriol.

[36] Yildiz FH, Schoolnik GK (1998). Role of RpoS in stress survival and virulence of *Vibrio cholerae*.. J Bacteriol.

[37] Song T, Mika F, Lindmark B, Liu Z, Schild S (2008). A new *Vibrio cholerae* sRNA modulates colonization and affects release of outer membrane vesicles.. Mol Microbiol.

[38] Thelin KH, Taylor RK (1996). Toxin-coregulated pilus, but not mannose-sensitive hemagglutinin, is required for colonization by *Vibrio cholerae* O1 El Tor biotype and O139 strains.. Infect Immun.

[39] Bolivar F, Rodriguez RL, Greene PJ, Betlach MC, Heyneker HL (1977). Construction and characterization of new cloning vehicles. II. A multipurpose cloning system.. Gene.

[40] Aiba H, Fujimoto S, Ozaki N (1982). Molecular cloning and nucleotide sequencing of the gene for *E. coli* cAMP receptor protein.. Nucleic Acids Res.

[41] Donnenberg MS, Kaper JB (1991). Construction of an *eae* deletion mutant of enteropathogenic *Escherichia coli* by using a positive-selection suicide vector.. Infect Immun.

[42] Skorupski K, Taylor RK (1996). Positive selection vectors for allelic exchange.. Gene.

[43] Valeru SP, Rompikuntal PK, Ishikawa T, Vaitkevicius K, Sjoling A (2009). Role of melanin pigment in expression of *Vibrio cholerae* virulence factors.. Infect Immun.

